# Posttraumatic *Bacillus cereus* Endophthalmitis: Clinical Characteristics and Antibiotic Susceptibilities

**DOI:** 10.1155/2021/6634179

**Published:** 2021-03-17

**Authors:** Feng Mei, Jiaqi Lin, Manli Liu, Yao Yang, Xiaofeng Lin, Fang Duan

**Affiliations:** Zhongshan Ophthalmic Center, State Key Laboratory of Ophthalmology, Sun Yat-Sen University, Guangzhou 510060, China

## Abstract

**Purpose:**

To report the clinical characteristics, antibiotic susceptibilities, and visual outcomes of patients with posttraumatic endophthalmitis caused by *Bacillus cereus*.

**Methods:**

In this retrospective, noncomparative case series, the medical records of eyes with culture-proven *Bacillus cereus* endophthalmitis treated from January 2016 to December 2019 at a referral center were reviewed. Clinical features, antibiotic susceptibilities, and visual outcomes were assessed.

**Results:**

A total of 19 eyes of 19 patients were identified. Three patients progressed to orbital cellulitis. Vitrectomy was performed in 13 eyes, and 11 required silicone oil tamponade. Finally, seven eyes underwent silicone oil removal surgery during follow-up. Only two patients retained a visual acuity better than FC. Four patients underwent evisceration, and three patients had NLP. The cultured *Bacillus cereus* was sensitive to levofloxacin, ofloxacin, tobramycin, and neomycin at 100%.

**Conclusions:**

The visual outcomes of posttraumatic *Bacillus cereus* endophthalmitis were generally poor regardless of the prophylactic and therapeutic measures administered. Vitrectomy combined with silicone oil tamponade could help to save the eyeball. *Bacillus cereus* has a good susceptibility to ofloxacin, levofloxacin, tobramycin, and neomycin; therefore, fluoroquinolones and aminoglycosides can be used to treat *Bacillus cereus* infection.

## 1. Introduction

Ocular trauma is a common eye disease and one of the most important causes of unilateral blindness, particularly in developing countries [1]. An estimated 55 million eye injuries occur annually, of which 19 million result in vision loss or blindness [2]. Posttraumatic endophthalmitis is an important complication of open globe injury. The prevalence of posttraumatic endophthalmitis has been reported to vary widely, from 0.9% to 11.9% [3–6], and may be much higher, from 6.9% to 30%, in intraocular foreign body (IOFB) injuries [7–9]. In addition, IOFB is reported to be present in 43% of eyes diagnosed with traumatic endophthalmitis [10]. The spectrum of causative organisms of posttraumatic endophthalmitis varies in different regions. Bacteria account for approximately 80–90% of culture-positive cases, and gram-positive cocci are the most common isolates, followed by gram-negative bacilli and gram-positive bacilli [11, 12]. *Bacillus cereus*, as gram-positive bacilli, is a group of uncommon but aggressive pathogens and accounts for approximately 1.5–10.7% of infectious endophthalmitis cases [12–15]. Furthermore, *Bacillus* spp. was the most common isolate and accounted for 17.1% of posttraumatic endophthalmitis cases [16]. In a review, the authors summarized 94 posttraumatic endophthalmitis cases involving an IOFB from 12 different studies; 34% of cases were infected by *Bacillus* spp. as either a single isolate or part of a polymicrobial infection [17]. Of them, *Bacillus cereus* was the most common isolate. Therefore, *Bacillus cereus* can be considered a very important pathogen in posttraumatic endophthalmitis.

The prognosis of posttraumatic *Bacillus cereus* endophthalmitis is poor despite vigorous treatment. In earlier studies, most cases of *Bacillus cereus* endophthalmitis resulted in enucleation [18–23]. In recent years, vitrectomy has been used to treat *Bacillus cereus* endophthalmitis. For example, Pan et al. reported 15 patients with *Bacillus cereus* endophthalmitis treated by vitrectomy with or without endoscopy assistance; however, four of them underwent enucleation [24]. There is no doubt that early application of sensitive antibiotics is helpful in controlling infections. Nevertheless, antibiotic resistance of *Bacillus cereus* was only reported in the 1990s [18, 25]; however, antibiotic resistance changed over time. Therefore, it is necessary to understand the antibiotic resistance that has developed in recent years for the successful early application of antibiotics, which plays an important role in the treatment of endophthalmitis.

The purpose of the current study was to review the characteristics of the clinical manifestations of posttraumatic *Bacillus cereus* endophthalmitis and the results of drug sensitivity in intraocular cultured isolates. These findings will provide a reference for guiding the use of antibiotics to treat *Bacillus cereus* infection.

## 2. Methods

### 2.1. Population

A retrospective review was conducted on patients with culture-positive *Bacillus cereus* endophthalmitis at the Zhongshan Ophthalmic Center (ZOC), Sun Yat-sen University (Guangzhou, China) between January 2016 and December 2019. This study was performed in compliance with the principles of the Declaration of Helsinki and was approved by the Institutional Ethics Committee of Zhongshan Ophthalmic Center, Sun Yat-sen University. Medical history, demographic data, laboratory results, and treatment records, including surgical records, were collected and analyzed. Vitreous opacity was detected by ocular B-mode ultrasonography scan. All the patients with open globe injuries routinely received prophylactic intravenous cefuroxime during primary repair. When patients were clinically diagnosed with endophthalmitis, the intravenous antibiotics were changed to vancomycin and ceftazidime. Intravitreal injections of vancomycin or cefuroxime were used in patients with strong suspicion of endophthalmitis. Vitrectomy was performed when the infection progressed rapidly and involved the posterior segment of the eyeball. Silicone oil tamponade was combined with vitrectomy when the retinal detachment or retinopathy occurred. Postoperatively, all the patients were continued on intravenous antibiotic for 5–7 days. The minimum follow-up time was three months.

### 2.2. Pathogen Isolation and Identification

The aqueous humor was aspirated from the anterior chamber through the limbus with a needle on a 1 mL syringe. Vitreous specimens were collected through the pars plana. Corneal specimens were collected by scraping the base and edge of the corneal ulceration with a platinum spatula. The specimens were inoculated into bacterial media, including blood agar or chocolate agar. Bacterial isolates were identified using the automated system (VITEK 2 compact BioMérieux, Inc., Marcy l'Étoile, France).

### 2.3. Antibiotic Susceptibility Test

Antibiotic susceptibility testing was performed with a minimum inhibitory concentration assay for beta-lactam antibiotics (penicillin, cefoxitin, and cefuroxime), fluoroquinolones (ofloxacin and levofloxacin), aminoglycosides (tobramycin, neomycin, and amikacin), and azithromycin. Antibiotic susceptibility was determined in accordance with the methods of the Clinical and Laboratory Standards Institute. Bacterial susceptibilities were recorded as “resistant,” “intermediate,” or “sensitive.” For the purpose of this study, being “intermediate” and being “sensitive” were both considered sensitive.

## 3. Results

A total of 19 patients (19 eyes) had culture-proven *Bacillus cereus* endophthalmitis in this retrospective study.  Table 1 presents the clinical data recorded for each patient. The mean age of the patients was 33.1 ± 21.7 years (range: 2–61 years), and all patients were male. School-age children (6–12 years old) and 45- to 56-year-old patients constituted 31.6% and 52.6% of the total, respectively. The most common occupation type was worker (11/19, 57.9%).

The types of injury included penetrating injuries, rupture, and foreign body injuries of the eyeball, and these traumas were mainly caused by the use of lawn mowers, industrial metal appliances, sharps, and plant scratches. Intraocular foreign bodies were present in nine cases (47.3%), two of which were restricted to the anterior chamber, while seven were in the posterior segment. In terms of the nature of the IOFB, it was metallic in six cases, glass in one case, an eyelash in one case, and a nonmetallic object in one case. Five cases occurred in soil-related activities, and four of them were related to the use of lawn mowers. The onset and deterioration of *Bacillus cereus* endophthalmitis occurred rapidly in all cases, accompanied by severe chemosis, pain, proptosis, corneal ring abscess, and pus in the anterior chamber. The onset of endophthalmitis occurred within 24 hours of the trauma in 16 patients and within 48 hours in three patients. In addition, three cases developed to orbital cellulitis.

Presenting and final visual acuity were unavailable in two patients because of their young age at the time of injury (2 and 6 years old). Only one patient had a visual acuity of 20/400, three patients had no light perception, and the rest of the patients had visual acuity ranging from light perception to 20/1000. Thirteen patients underwent vitrectomy, and eleven eyes were filled with silicone oil. Among them, seven eyes underwent silicone oil removal surgery. Only two eyes retained a visual acuity better than FC after the removal of silicone oil. The visual acuity of case number 14 was unavailable because of young age (2 years). The final visual acuity ranged from LP to FC in the remaining four cases. After vigorous treatments, enucleation was finally performed in four cases, and an ocular prosthesis was implanted.

Table 2 shows that the most common places were the workplace and grassland, and most of them (17/19) were caused by their own careless operation. The length of the wound varied from 2 to 10 mm, and most of them are zone I injuries. In 11 cases, the apparent length of the wound was less than 3 mm. In all cases, retinal detachment developed in nine patients, and seven of them also had choroid detachment. Three workers presented orbital cellulitis, one patient underwent evisceration, and two patients were administered sensitive antibiotics to control inflammation. The final visual acuity of these two eyes was NLP.

Table 3 presents the results of the drug sensitivity test, in which the penicillin resistance rate was 100%. Azithromycin and amikacin were available for the isolates. *Bacillus cereus* was highly sensitive to tobramycin, neomycin, ofloxacin, and levofloxacin.

## 4. Discussion

In the present study, the medical records and visual outcomes of 19 patients with posttraumatic *Bacillus cereus* endophthalmitis were reviewed. We found that the outcomes of posttraumatic *Bacillus cereus* were still poor, even after vigorous treatment. Four patients underwent evisceration, and three patients had NLP. This highlights the importance of extensive research for improving the treatment strategies for ocular conditions. In addition, the cultured *Bacillus cereus* was still 100% sensitive to levofloxacin, ofloxacin, tobramycin, and neomycin.

*Bacillus cereus* endophthalmitis is a devastating intraocular infection primarily associated with posttraumatic injuries, especially in patients with IOFBs [12–17]. The majority of these infections always result in substantial vision loss within 12–48 hours [26]. In the current study, all the patients suffered from typical eye pain and severe loss of vision within 48 hours, with 16 patients experiencing these symptoms within 24 hours and three within 24 hours, which is consistent with many previous studies [18, 27–29], illustrating the virulence of *Bacillus cereus* to the function of the eyeball, and it is difficult to improve the function after treatment. In experimental rabbit *Bacillus cereus* endophthalmitis, early intravitreal treatment at two or four hours after the infection with vancomycin or gatifloxacin improved the therapeutic outcome [30], and early vitrectomy had a therapeutic benefit at four hours after the infection [31]. In addition, these two studies indicated that a delay in treatment beyond six hours after the infection led to a substantial loss of retinal function. Therefore, the window of therapeutic intervention in *Bacillus cereus* endophthalmitis is quite narrow, and rapid identification and proper treatment should be initiated as early as possible.

Many previous reports have shown that the outcomes for patients with posttraumatic *Bacillus cereus* endophthalmitis are very poor and often lead to complete vision loss with limited potential for the restoration of useful vision. Some patients with *Bacillus cereus* endophthalmitis undergo enucleation [18–23]. In our study, at the final postoperative follow-up, three patients had no light perception, only one patient had a visual acuity of 20/400, and the rest of the patients had a visual acuity ranging from light perception to 20/1000, except the two children who could not be examined. Thirteen patients underwent vitrectomy, and 11 of them required silicone oil tamponade. Finally, seven eyes were preserved after silicone oil removal surgery. Only two of these eyes retained visual acuity better than FC. The rate of evisceration in our case series was relatively lower than that in previous studies, which indicated that vitrectomy combined with silicone oil tamponade could help preserve the eyeball. Three patients could not undergo vitrectomy because of severe corneal opacity in our series. Previous reports have shown that posttraumatic *Bacillus cereus* endophthalmitis can cause complete corneal opacification within 24 hours of injury [32]. Pan et al. recommended endoscopy-assisted vitrectomy as an alternative treatment for *Bacillus cereus* endophthalmitis [24].

In our study, nine patients had IOFBs, and most of them had metallic foreign bodies, which is consistent with a previous study of *Bacillus cereus* endophthalmitis [24]. A previous study reported that *Bacillus* species was the most common pathogen in posttraumatic endophthalmitis cases involving IOFB [17], which indicated that posttraumatic endophthalmitis with *Bacillus* species is strongly associated with the presence of an IOFB. In addition, four cases were related to the use of lawn mowers and injured by soil-contaminated IOFBs, which is consistent with a previous study [26]. *Bacillus* species spores are abundant in soil, which may explain why *Bacillus* endophthalmitis occurs relatively more frequently in cases of soil-contaminated IOFBs.

Administration of antibiotics is a mainstay in the management of endophthalmitis. In 1988, Weber et al. reported that *Bacillus cereus* was 100% susceptible to vancomycin and imipenem and more than 90% susceptible to chloramphenicol, ciprofloxacin and gentamicin [25]. Meanwhile, *Bacillus cereus* is resistant to penicillin, oxacillin, cefuroxime, and cefotaxime, consistent with our study. Intravitreal vancomycin (1 mg in 0.1 mL normal saline) and intravenous vancomycin can provide good therapeutic effects against *Bacillus cereus* and are still the recommended treatments. In our study, *Bacillus cereus* also showed good susceptibility to ofloxacin, levofloxacin, tobramycin, and neomycin; therefore, fluoroquinolones and aminoglycosides can also be used to treat *Bacillus cereus* infection.

The limitations of this study included its retrospective nature and relatively small size. The youngest subject in our series was two years old, limiting the full assessment of visual outcome. Finally, information regarding the antibiotic resistance of some gram-positive bacteria, including vancomycin and fourth-generation quinolones, was lacking. Nevertheless, our study provides valid data on the treatment of *Bacillus cereus* endophthalmitis.

In conclusion, although vitrectomy has been widely used, the visual outcome of posttraumatic *Bacillus cereus* is still very poor. The rate of evisceration in our case series was relatively lower than that reported in previous studies. *Bacillus cereus* was found to be highly sensitive to tobramycin, neomycin, ofloxacin, and levofloxacin.

## Figures and Tables

**Table 1:** Demographic characteristics and outcomes of the 19 patients with *B. cereus* endophthalmitis.

Case no./age/sex	Job	Eye	Cause of trauma	Onset time (day)	Initial VA	Character of IOFB	Culture	Surgery	Intraocular tamponade	Final VA
1/55/M	Worker	OS	Foreign body when digging the ground	1	LP	Metal	Vitreous	PPV	Silicone oil	HM
2/51/M^*∗*^	Worker	OS	Nail	1	NLP	No	Aqueous	Evisceration	—	Ocular prosthesis
3/45/M	Worker	OD	Nail	1	HM	Metal	Vitreous	PPV	BBS	HM
4/50/M	Worker	OS	Foreign body when hammering	1	NLP	Metal	Aqueous	Evisceration	—	Ocular prosthesis
5/7/M	Student	OD	Slingshot	2	LP	No	Vitreous	PPV	Silicone oil	FC#
6/45/M	Peasantry	OD	Foreign body when mowing grass	1	NLP	Metal	Vitreous	PPV	BBS	NLP
7/6/M	No job	OD	Reed pole	2	LP	No	Vitreous	Evisceration	—	Ocular prosthesis
8/8/M	Student	OS	Wire	2	HM	No	Vitreous	PPV	Silicone oil	20/1000#
9/48/M	Worker	OS	Foreign body when mowing grass	1	LP	Glass	Vitreous	PPV	Silicone oil	FC
10/56/M	Worker	OS	Foreign body when mowing grass	1	LP	No	Vitreous	Evisceration	—	Ocular prosthesis
11/6/M	No job	OD	Scissors	1	HM	No	Vitreous	PPV	Silicone oil	20/400#
12/50/M	Worker	OD	Foreign body when mowing grass	1	LP	Metal	Vitreous	PPV	Silicone oil	LP#
13/47/M^*∗*^	Worker	OD	Fire extinguisher explosion	1	HM	Nonmetallic	Vitreous	—	—	NLP
14/6/M	No job	OS	Wooden stick	1	—	No	Vitreous	PPV	Silicone oil	—#
15/2/M	No job	OD	Wire	1	—	Cilia	Vitreous	PPV	Silicone oil	—
16/61/M	Worker	OS	Foreign body when hammering	1	LP	Metal	Vitreous	PPV	Silicone oil	LP
17/7/M	Student	OS	Wooden stick	1	LP	No	Vitreous	PPV	Silicone oil	HM#
18/52/M^*∗*^	Worker	OD	Board	1	NLP	No	Cornea	—	—	NLP
19/27/M	Worker	OS	Stone	1	HM	No	Vitreous	PPV	Silicone oil	HM#

^*∗*^Patients with orbital cellulitis. #Patients with silicone oil removal. VA: visual acuity; NLP: no light perception; LP: light perception; HM: hand movement; FC: figure count; PPV: pars plana vitrectomy.

**Table 2:** Clinical characteristics of the 19 patients with *B. cereus* endophthalmitis.

Variable	*N* (%)
Place of injury	
Home	2/19, 10.5
Workplace	8/19, 42.1
Grassland	6/19, 31.6
Restaurant	2/19, 10.5
School	1/19, 5.3

Injure condition	
Injured by others	2/19, 10.5
Injured by themselves	17/19, 89.5

Location of wound	
Zone I	17/19, 89.5
Zone II	2/19, 10.5

Size of wound	
≤3 mm	11/19, 57.9
4 mm–6 mm	6/19, 31.6
≥6 mm	2/19, 10.5

Corneal ulcer	3/19, 15.8
Hypopyon	12/19, 63.2
Traumatic cataract	19/19, 100
Serious vitreous opacity	19/19, 100
Retinal detachment	9/19, 47.4
Choroid detachment	7/19, 36.8
Orbital cellulitis (*n*)	3/19, 15.8

**Table 3:** Antibacterial resistance of cultured *Bacillus cereus.*

Antibiotic	Antibiotic resistance rate (*n*, %)
Beta-lactam antibiotics	
Penicillin	19/19, 100.0
Cefuroxime	17/19, 89.5
Cefoxitin	13/19, 68.4

Macrolides antibiotics	
Azithromycin	1/8, 12.5

Aminoglycosides antibiotics	
Amikacin	2/19, 10.5
Tobramycin	0/18, 0.0
Neomycin	0/7, 0.0

Quinolone antibiotics	
Levofloxacin	0/19, 0.0
Ofloxacin	0/19, 0.0

## Data Availability

All the data used to support the findings of this study are included within the article and are available from the corresponding author upon reasonable request.
